# The relationship between healthy sleep patterns and the risk of scoliosis: a large prospective cohort study

**DOI:** 10.3389/fnins.2026.1839503

**Published:** 2026-06-17

**Authors:** Wanyue Li, Shuxiao Ma, Mei Hu, Yanzhao Hao, Ying Li, Xiguo Cai, Weisheng Zhuang, Ruihua Sun

**Affiliations:** 1 Henan Provincial People’s Hospital and People’s Hospital of Zhengzhou University, Zhengzhou, China; 2 Department of Rehabilitation, Henan Provincial People’s Hospital and People’s Hospital of Zhengzhou University, Zhengzhou, China

**Keywords:** cohort study, interaction effect, scoliosis, sleep pattern, UK Biobank

## Abstract

**Background:**

Currently, prospective evidence on how sleep habits specifically affect scoliosis is nearly nonexistent. We therefore sought to clarify the association between comprehensive sleep behavior patterns and the incidence of this disease.

**Methods:**

This study conducted a prospective cohort study based on the UK Biobank (UKB), including 408,870 participants who did not have scoliosis at baseline. We have constructed a comprehensive sleep scoring system that integrates the following five key indicators: sleep chronotype, sleep duration, insomnia, snoring, and daytime sleepiness. To evaluate the association between healthy sleep patterns and the risk of scoliosis, we conducted a statistical analysis using the Cox proportional hazards regression model.

**Results:**

Over a mean follow-up of 15.82 years, 2,976 incident cases of scoliosis were recorded (0.73%). The 408,870 participants who were free of scoliosis at baseline had a mean age of 56.48 years. Distribution of the healthy sleep score was as follows: 9,939 participants (2.43%) had 0–1 of the five healthy sleep behaviors, 46,175 (11.29%) had 2 behaviors, 115,094 (28.15%) had 3 behaviors, 150,287 (36.76%) had 4 behaviors, and 87,375 (21.37%) had all five. In multivariable models, each 1-point increase in the healthy sleep score was associated with a 10% lower risk of scoliosis [hazard ratio (HR) = 0.90; 95% confidence interval (CI) = 0.87, 0.93]. Compared with the 0–1 score group, the multivariable-adjusted HR (95% CI) for participants with a score of 5 was 0.64 (0.52, 0.80). In subgroup analyses, the inverse association between sleep score and scoliosis risk persisted among participants without diabetes but was absent among those with diabetes (*p*_*interaction*_ < 0.05).

**Conclusion:**

Cohort analysis results confirm that adherence to a healthy sleep-behavior pattern significantly reduces the risk of scoliosis.

## 1 Introduction

Scoliosis is a complex three-dimensional spinal deformity marked by lateral curvature in the coronal plane, typically accompanied by vertebral rotation and altered sagittal alignment (Hoy et al., 2012; Weinstein et al., 1981). The condition not only alters physical appearance but can also cause chronic back pain, cardiopulmonary dysfunction, neurological impairment, and substantial psychological distress, all of which reduce quality of life and impose a heavy socioeconomic burden (Kobayashi et al., 2023; Nikaido et al., 2025). Although Adolescent Idiopathic Scoliosis (AIS) is the most widely studied type, new-onset or progressive Scoliosis in the adult population, especially Degenerative Scoliosis, Its prevalence rate has risen significantly with the global aging of the population, posing an increasingly severe public health challenge (Quintero Santofimio et al., 2023). At present, the etiology and pathogenesis of scoliosis have not been fully clarified. It is generally believed that it is the result of the complex interaction of multiple factors such as genetics, hormones, the nervous system, biomechanics and environmental factors (Kuang et al., 2025). Therefore, identifying and intervening in the modifiable risk factors among them is crucial for formulating effective primary prevention strategies.

Sleep is an essential physiological process necessary for sustaining life activities and plays a pivotal role in overall health. A healthy sleep pattern is a multidimensional concept that encompasses not only adequate sleep duration but also sleep chronotype, the absence of insomnia, and sufficient daytime energy (He et al., 2022; Pu et al., 2024; Wan et al., 2022). Research indicates that poor sleep habits, including sleep deprivation, circadian rhythm disorders, and sleep-related breathing disorders, serve as independent risk factors for numerous chronic non-communicable diseases, such as cardiovascular diseases, type 2 diabetes, obesity, neurodegenerative diseases, and certain cancers (Dashti et al., 2019; Fan et al., 2020; Sambou et al., 2021). Research shows that, even with optimal pillow support, variations in sleeping position markedly alter neck muscle activity and can precipitate neck and shoulder pain (Won-Hwee and Min-Seok, 2017). Poor sleep quality also correlates with a higher incidence of low back pain and with heightened pain sensitivity in patients with spinal deformities (Yakut et al., 2022). The onset and progression of these diseases are frequently linked to several core physiological pathways regulated by sleep, including endocrine and hormonal regulation, immune and inflammatory responses, metabolism, and cellular repair processes (Jurado-Fasoli et al., 2018; Sambou et al., 2021).

Given that sleep significantly influences the health of bones, muscles, and the nervous system, the potential link between sleep and spinal health has garnered increasing attention from the academic community. Existing research primarily investigates the sleep conditions of patients diagnosed with scoliosis. Most of these studies employ cross-sectional designs and consistently demonstrate that the prevalence of issues such as poor sleep quality, insomnia, and daytime sleepiness among scoliosis patients is markedly higher than that in healthy individuals (Ugur et al., 2023). However, these associations likely reflect the adverse effects of the disease on sleep, including chronic pain from spinal deformities and muscle imbalances, sleep apnea due to compromised respiratory function, and psychological stress stemming from appearance-related anxiety and functional limitations, all of which directly contribute to sleep disorders. Consequently, current research cannot establish whether sleep problems precede or follow scoliosis. The potential causal direction and temporal sequence therefore remain unresolved scientific questions.

To date, there has been a significant deficiency of large-scale, long-term follow-up prospective cohort studies globally that directly investigate the relationship between healthy sleep patterns and the risk of new-onset scoliosis. This evidence gap restricts our comprehensive understanding of the risk factors that may influence scoliosis and impedes the development of effective prevention strategies. The UK Biobank (UKB), a large-scale prospective cohort study with approximately 500,000 participants, provides detailed lifestyle information and long-term linkage to national health records, thereby presenting a unique opportunity and valuable data resources to address this scientific issue (Bycroft et al., 2018; Cribb et al., 2023; Sudlow et al., 2015). Consequently, this study aimed to utilize the UKB database to prospectively examine, for the first time, a comprehensive healthy sleep pattern score constructed from five key dimensions: sleep chronotype, sleep duration, daytime sleepiness, insomnia, and snoring (Trabelsi et al., 2021). We will assess the association of these factors with the risk of new-onset scoliosis in middle-aged and elderly populations. We hypothesized that adherence to healthier and more multidimensional sleep habits will be significantly correlated with a reduced risk of scoliosis in the future.

## 2 Materials and methods

### 2.1 Study design

This study utilized data from the UKB, a cohort that successfully recruited over 500,000 ordinary adults aged 38–70 between 2006 and 2010. The UKB provides information on sleep and various health-related factors, collected through baseline and subsequent touchscreen questionnaires, oral interviews, biological samples, and body measurements. All participants underwent standardized physical examinations and provided high-quality biological samples. Following this, the research team conducted continuous long-term longitudinal follow-up and updates on the aforementioned key indicators (Sudlow et al., 2015; Yévenes-Briones et al., 2022).

**FIGURE 1:**
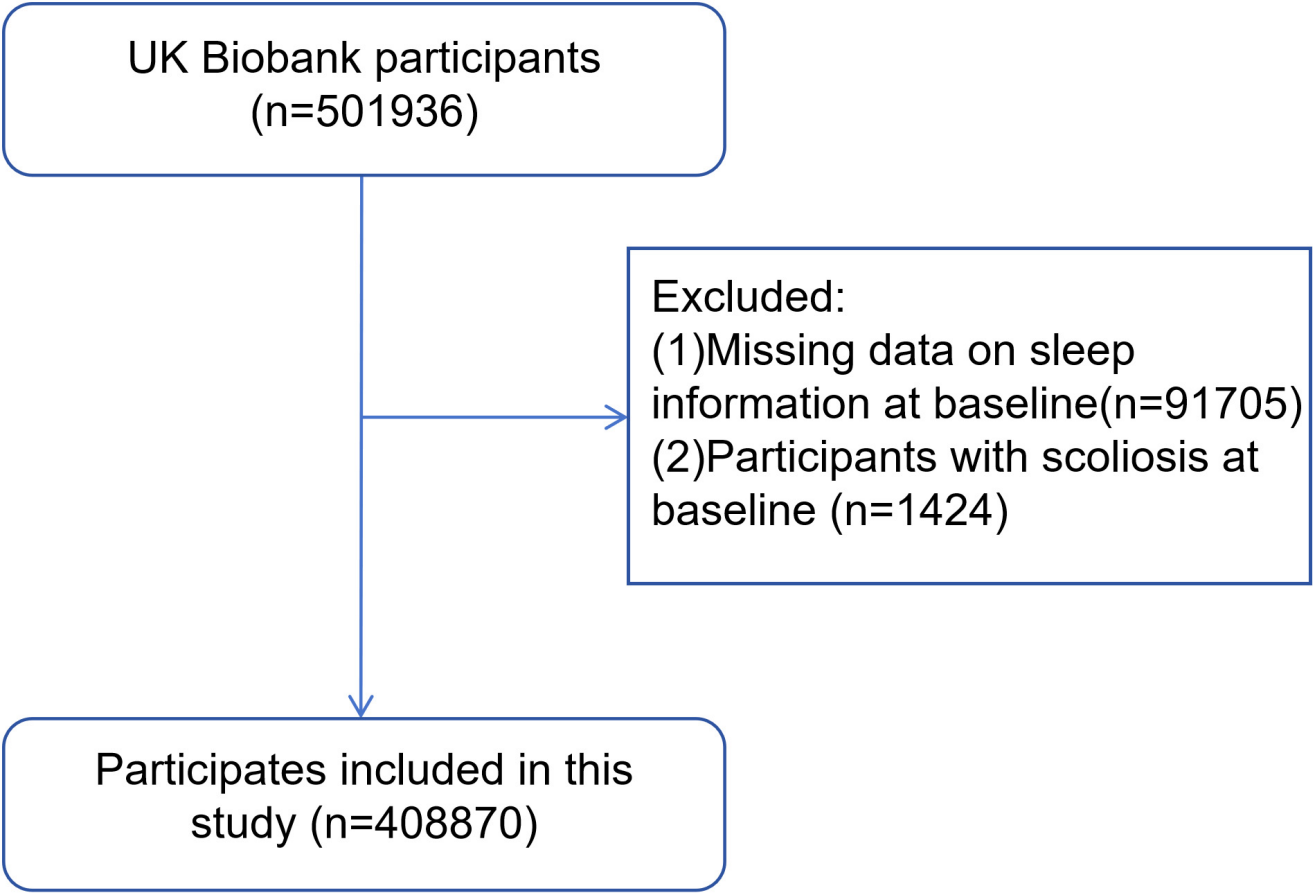
Flowchart for the participants who were included in the analysis.

This study received official approval from the National Information Management Board for Health and Social Care in England and Wales, the Scottish Community Health Index Advisory Group, and the Northwest Multicenter Research Ethics Committee. Clinical trial number: not applicable. All participants provided written informed consent (Sudlow et al., 2015). During the data analysis stage, we excluded the following individuals who did not meet the inclusion criteria: those with missing data in each dimension of sleep scores (*n* = 91,705) and participants who already had scoliosis at baseline (*n* = 1,424). The final valid sample size used for statistical analysis was 408,870 (Figure 1).

### 2.2 Assessment of exposure

At baseline, data were collected on sleep chronotype, sleep duration, daytime sleepiness, and insomnia. Information on various sleep behaviors, including snoring, was also gathered (for detailed questionnaire content, see Supplementary Table 1). This study defines healthy sleep patterns as a composite of behaviors encompassing five dimensions. In accordance with established standards in the literature, we categorize the aforementioned behaviors into two groups: “low-risk” and “high-risk.” Healthy sleep behaviors were defined as follows: an early chronotype, characterized as a “morning” person rather than an “evening” person; a sleep duration of 7–8 h per day; infrequent insomnia symptoms, categorized as “sometimes” or “never/rarely;” absence of self-reported snoring; and infrequent daytime sleepiness, also categorized as “sometimes” or “never/rarely.” A score of 1 was assigned to each of these criteria, while a score of 0 was given to all other criteria (Dergaa et al., 2022). The final composite healthy sleep score, which ranges from 0 to 5, represents the sum of the scores for each indicator, with higher scores reflecting better sleep quality (Fan et al., 2020; Shi et al., 2022; Zou et al., 2025).

### 2.3 Assessment of outcomes

In this study, the occurrence of scoliosis served as the primary outcome measure, defined in accordance with the Tenth Revision of the International Classification of Diseases (ICD-10). Specifically, cases were identified using ICD-10 code M41, which includes M41.0, M41.1, M41.2, M41.3, M41.4, M41.5, M41.8, and M41.9 (Kolenkiewicz et al., 2018). The follow-up duration for each participant was determined by the interval from the date of enrollment to the earliest occurrence of one of the following events: diagnosis of scoliosis, death, loss to follow-up, or the follow-up deadline of August 31, 2025.

### 2.4 Assessment of covariates

To mitigate the influence of specific confounding factors, we considered several baseline covariates: age, sex (male or female), ethnicity (white or other), education level (university/college degree or other), Townsend Deprivation Index (TDI), household income, body mass index (BMI), smoking status (never, previous, or current), alcohol consumption (never, previous, or current), diabetes status (yes or no), hypertension status (yes or no), and metabolic equivalent (MET) time per week, defined as “summed MET minutes per week for all activity.”

### 2.5 Statistical analyses

Multivariate imputation by chained equations with random forest was used for imputing missing data (Shah et al., 2014). The baseline characteristics of the participants were described using the average or percentage based on the distribution of the healthy sleep score. Given that the number of individuals scoring 0 and 1 in the healthy sleep score is relatively small, we combined them into a single category for analysis. To evaluate the association between sleep scores and the risk of scoliosis, we constructed a Cox proportional hazards regression model. This model takes the scores into account both as ordered categorical variables and as continuous variables (for each additional point). Ultimately, in addition to the unadjusted model, we fitted two corrected models: Model 1 controls age, sex, ethnicity, household income, educational level, and Townsend deprivation index; Model 2 further adjusted the body mass index, smoking status, alcohol status, diabetes, hypertension, and MET time per week on this basis. The distribution of MET hours/week was right-skewed. To evaluate the Cox model’s linearity assumption for this covariate, we performed a restricted cubic spline (RCS) analysis with 3 knots. The test for nonlinearity was significant (*P* = 3.56 × 10-^5^), indicating a J-shaped association (Supplementary Figure 1). Consequently, MET minutes/week was entered into the Cox model as a categorical variable reflecting the observed J-shaped pattern: ≤ 49, < 49 ≤ 149, < 149 ≤ 199, and < 199 MET-hours/week.

Subgroup analyses were performed based on age ( < 60 years old, ≥ 60 years old), sex (male, female), educational level (College/University, other), diabetes status (yes or no), and hypertension status (yes or no) to assess potential effects of alterations in MET time per week ( < 40 h, ≥ 40 h). The interaction effect of these stratified factors on the risk of scoliosis and the healthy sleep score was evaluated using the likelihood ratio test, which compared models with and without a cross-product term. Individual healthy sleep behaviors and healthy sleep scores were assessed to calculate the proportion of the population attributable to unhealthy sleep patterns, expressed as the population attributable risk percentage (PAR%). We estimated PAR% using the formula p * (HR − 1)/[1 + p * (HR − 1)], where HR represents the relevant fully adjusted hazard ratio for the participants and p denotes the proportion of participants who do not belong to the healthy group.

This study performed a series of sensitivity analyses to assess the robustness of the findings. First, to mitigate the concern of reverse causality, we excluded individuals who exhibited symptoms of spinal curvature within 1, 2, 5, and 10 years during the follow-up period. Second, to control for demographic confounding factors, we retained only white participants and excluded samples from other racial groups. Third, recognizing that the COVID-19 pandemic could disrupt health behaviors, we further excluded individuals enrolled after the outbreak. Fourth, we constructed a weighted healthy sleep score based on five sleep behaviors, with the weights derived from the adjusted hazard ratio (HR) of the Cox proportional hazards model. Fifth, we conducted multiple imputation for missing sleep data and re-ran the primary analyses to assess the robustness of our findings. Finally, to address the potential risk of death competition, we conducted a sensitivity analysis using the Fine-Gray sub-distribution risk model (Schuster et al., 2020), treating death as a competing event, in order to assess the robustness of our research results to the assumption of competition risk.

All statistical analyses were conducted using the R package (version 4.4.2), where a *P-*value < 0.05 was considered statistically significant.

## 3 Results

### 3.1 Baseline characteristics

The average follow-up duration for the study population was 15.82 years (SD 2.66). Table 1 details the baseline characteristics of the participants, categorized by Healthy Sleep score. Among the 408,870 individuals, the mean age was 56.48 years (SD 8.09), with 224,753 females, representing 55.0% of the cohort. The distribution of sleep scores at baseline was as follows: 9,939 participants (2.43%) scored 0–1 points, 46,175 (11.29%) scored 2 points, 115,094 (28.15%) scored 3 points, 150,287 (36.76%) scored 4 points, and 87,375 (21.37%) scored 5 points. Notably, participants with higher Healthy Sleep scores exhibited longer activity durations, higher incomes, a reduced prevalence of diabetes and hypertension, and lower rates of alcohol consumption and smoking. Additionally, they had lower BMI and TDI (Table 1). We performed a comprehensive comparison of baseline characteristics between excluded and included participants, revealing systematic differences in age, socioeconomic status, and health indicators (Supplementary Table 2).

**TABLE 1:** Baseline characteristics according to healthy sleep score in the cohort.

Characteristics	Overall	Healthy sleep score					*P-*value
		0–1	2	3	4	5	
	(*N* = 408,870)	(*N* = 9,939)	(*N* = 46,175)	(*N* = 115,094)	(*N* = 150,287)	(*N* = 87,375)	
Age	56.5 (8.09)	56.6 (7.74)	56.7 (7.83)	56.7 (7.97)	56.4 (8.15)	56.2 (8.31)	< 0.001
Sex Female	224,753 (55.0%)	5,067 (51.0%)	24,242 (52.5%)	59,393 (51.6%)	81,054 (53.9%)	54,997 (62.9%)	< 0.001
Male	184,117 (45.0%)	4,872 (49.0%)	21,933 (47.5%)	55,701 (48.4%)	69,233 (46.1%)	32,378 (37.1%)	
BMI	27.4 (4.77)	30.1 (5.87)	28.7 (5.28)	27.9 (4.83)	27.1 (4.54)	26.2 (4.26)	< 0.001
Townsend deprivation index	−1.41 (3.03)	−0.582 (3.38)	−1.05 (3.20)	−1.32 (3.08)	−1.51 (2.97)	−1.62 (2.91)	< 0.001
MET hours/week	29.8 (45.8)	23.6 (43.6)	26.1 (43.8)	28.5 (45.4)	30.8 (45.7)	33.1 (46.8)	< 0.001
Ethnicity White people	377,669 (92.4%)	8,952 (90.1%)	42,343 (91.7%)	106,136 (92.2%)	139,035 (92.5%)	81,203 (92.9%)	< 0.001
Other people	31,201 (7.6%)	987 (9.9%)	3,832 (8.3%)	8,958 (7.8%)	11,252 (7.5%)	6,172 (7.1%)	
Education College/University	134,633 (32.9%)	2,433 (24.5%)	12,681 (27.5%)	35,631 (31.0%)	51,809 (34.5%)	32,079 (36.7%)	< 0.001
Other	274,237 (67.1%)	7,506 (75.5%)	33,494 (72.5%)	79,463 (69.0%)	98,478 (65.5%)	55,296 (63.3%)	
Smoking status Current	42,609 (10.4%)	1,789 (18.0%)	6,847 (14.8%)	13,888 (12.1%)	14,209 (9.5%)	5,876 (6.7%)	< 0.001
Previous	143,384 (35.1%)	3,903 (39.3%)	17,776 (38.5%)	42,257 (36.7%)	52,299 (34.8%)	27,149 (31.1%)	
Never	222,877 (54.5%)	4,247 (42.7%)	21,552 (46.7%)	58,949 (51.2%)	83,779 (55.7%)	54,350 (62.2%)	
Alcohol status current	377,610 (92.4%)	8,928 (89.8%)	42,400 (91.8%)	106,511 (92.5%)	139,389 (92.7%)	80,382 (92.0%)	< 0.001
Previous	14,102 (3.4%)	561 (5.6%)	1,951 (4.2%)	3,966 (3.4%)	4,829 (3.2%)	2,795 (3.2%)	
Never	17,158 (4.2%)	450 (4.5%)	1,824 (4.0%)	4,617 (4.0%)	6,069 (4.0%)	4,198 (4.8%)	
Household income Less than 18,000	89,831 (22.0%)	3,151 (31.7%)	12,274 (26.6%)	26,319 (22.9%)	30,669 (20.4%)	17,418 (19.9%)	< 0.001
18,000–30,999	105,344 (25.8%)	2,616 (26.3%)	12,155 (26.3%)	30,143 (26.2%)	38,362 (25.5%)	22,068 (25.3%)	
31,000–51,999	107,400 (26.3%)	2,306 (23.2%)	11,626 (25.2%)	30,029 (26.1%)	40,260 (26.8%)	23,179 (26.5%)	
52,000–100,000	83,872 (20.5%)	1,537 (15.5%)	8,206 (17.8%)	22,752 (19.8%)	32,211 (21.4%)	19,166 (21.9%)	
Greater than 100,000	22,423 (5.5%)	329 (3.3%)	1,914 (4.1%)	5,851 (5.1%)	8,785 (5.8%)	5,544 (6.3%)	
Diabetes Yes	20,940 (5.1%)	1,124 (11.3%)	3,499 (7.6%)	6,586 (5.7%)	6,747 (4.5%)	2,984 (3.4%)	
No	387,930 (94.9%)	8,815 (88.7%)	42,676 (92.4%)	108,508 (94.3%)	143,540 (95.5%)	84,391 (96.6%)	
Hypertension Yes	109,369 (26.7%)	4,010 (40.3%)	15,209 (32.9%)	33,627 (29.2%)	37,789 (25.1%)	18,734 (21.4%)	
No	299,501 (73.3%)	5,929 (59.7%)	30,966 (67.1%)	81,467 (70.8%)	112,498 (74.9%)	68,641 (78.6%)	
Chronotype evening person	152,315 (37.3%)	9,035 (90.9%)	32,965 (71.4%)	60,545 (52.6%)	49,770 (33.1%)	−	< 0.001
Morning person	256,555 (62.7%)	904 (9.1%)	13,210 (28.6%)	54,549 (47.4%)	100,517 (66.9%)	−	
Sleep duration 7–8 h/day	278,597 (68.1%)	431 (4.3%)	9,454 (20.5%)	58,288 (50.6%)	123,049 (81.9%)	−	< 0.001
Other	130,273 (31.9%)	9,508 (95.7%)	36,721 (79.5%)	56,806 (49.4%)	27,238 (18.1%)	−	
Frequent insomnia Yes	113,601 (27.8%)	9,454 (95.1%)	33,434 (72.4%)	48,732 (42.3%)	21,981 (14.6%)	−	< 0.001
No	295,269 (72.2%)	485 (4.9%)	12,741 (27.6%)	66,362 (57.7%)	128,306 (85.4%)	−	
Self-reported snoring Yes	152,057 (37.2%)	9,281 (93.4%)	31,601 (68.4%)	60,939 (52.9%)	50,236 (33.4%)	−	< 0.001
No	256,813 (62.8%)	658 (6.6%)	14,574 (31.6%)	54,155 (47.1%)	100,051 (66.6%)	−	
Frequent daytime dozing Yes	11,209 (2.7%)	3,177 (32.0%)	3,804 (8.2%)	3,166 (2.8%)	1,062 (0.7%)	−	< 0.001
No	397,661 (97.3%)	6,762 (68.0%)	42,371 (91.8%)	111,928 (97.2%)	149,225 (99.3%)	−	
Scoliosis No	405,894 (99.3%)	9,839 (99.0%)	45,772 (99.1%)	114,209 (99.2%)	149,275 (99.3%)	86,799 (99.3%)	< 0.001
Yes	2,976 (0.7%)	100 (1.0%)	403 (0.9%)	885 (0.8%)	1,012 (0.7%)	576 (0.7%)	
Year	15.8 (2.66)	15.5 (3.17)	15.6 (2.93)	15.8 (2.74)	15.9 (2.59)	15.9 (2.46)	< 0.001

The values for continuous variables are given as mean ± SD and values for categorical variables are given as numbers (percentage). BMI, body mass index; MET, metabolic equivalent.

### 3.2 Association between healthy sleep score and scoliosis events

During an average follow-up period of 15.82 years (SD 2.67), a total of 2,976 new cases of scoliosis (0.73%) were confirmed. The preliminary model indicates a significant negative correlation between the healthy sleep score and the risk of scoliosis (*P* trend < 0.001). This trend remained stable even after the introduction of additional covariates for multivariate correction, confirming that the risk of scoliosis decreases with improvements in sleep quality (*P* trend < 0.001). In the fully adjusted Model 2, participants with a healthy sleep score of 5 exhibited a significantly lower risk of developing scoliosis compared to those with a score of 0–1 (HR, 0.64; 95% CI 0.52–0.80; *P* trend < 0.001). Moreover, for each 1-point increase in the healthy sleep score, the risk of scoliosis decreased by 10% (HR, 0.90; 95% CI 0.87–0.93; *P* trend < 0.001) (Table 2).

**TABLE 2:** Risk of scoliosis associated with healthy sleep score.

Characteristics	Healthy sleep score					Per1-point increment	*P* for trend
	0–1	2	3	4	5		
Participants, n	9,939	46,175	115,094	150,287	87,375	−	−
Scoliosis, n	100	403	885	1,012	576	−	−
Follow-up period, month							
Means ± SD	15.5 (3.17)	15.6 (2.93)	15.8 (2.74)	15.9 (2.59)	15.9 (2.46)	−	−
Median (IQR)	16.3 [0.0438, 18.4]	16.4 [0.0137, 18.4]	16.4 [0.00822, 18.4]	16.4 [0.0247, 18.4]	16.5 [0.0301, 18.7]	−	−
Hazard ratio for incident scoliosis (95% CI)							
Crude model	Reference	0.86 (0.69, 1.07)	0.75 (0.61, 0.92)	0.65 (0.53, 0.80)	0.63 (0.51, 0.78)	0.90 (0.86, 0.93)	< 0.001
Adjusted model 1	Reference	0.85 (0.69, 1.06)	0.76 (0.62, 0.94)	0.68 (0.55, 0.83)	0.62 (0.50, 0.77)	0.89 (0.86, 0.93)	< 0.001
Adjusted model 2	Reference	0.86 (0.69, 1.08)	0.78 (0.63, 0.96)	0.70 (0.57, 0.86)	0.64 (0.52, 0.80)	0.90 (0.87, 0.93)	< 0.001

ACox proportional hazard model was conducted. Adjusted model 1, age, sex, ethnicity, household income, educational level, and Townsend deprivation index were adjusted for in this model; adjusted model 2, body mass index, smoking status, alcohol status, diabetes, hypertension, and Total weekly activity time additionally were adjusted for in this model. CI, confidence interval

**FIGURE 2:**
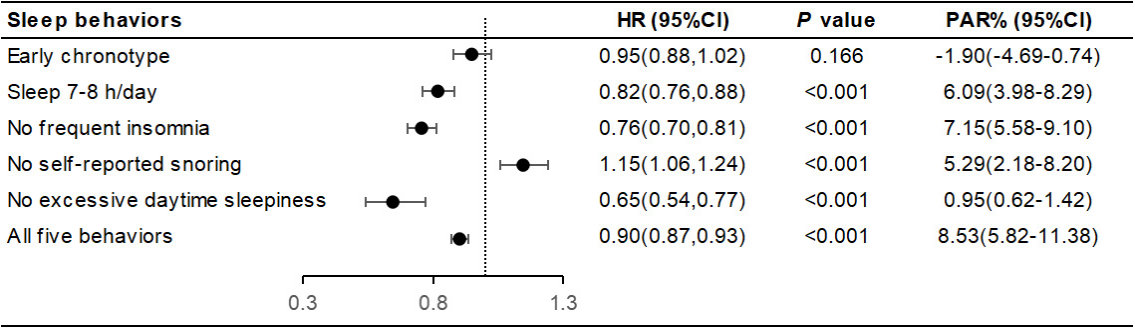
Associations of each healthy sleep behavior with the risk of scoliosis.

When an individual’s sleep behavior is regarded as a binary classification (high-risk and low-risk), having a sleep duration of 7–8 h per day, never/rarely experiencing insomnia, and having no frequent daytime sleepiness were associated with 18, 24, and 35% lower risks of scoliosis, respectively (Figure 2). The PAR% of scoliosis among participants with an overall healthy sleep score of 5 was 8.53% (95% CI 5.82, 11.38), indicating that if all participants were included in all five behaviors of the healthy sleep behavior group, approximately one-tenth of scoliosis events in this population would not occur (Figure 2). Supplementary Table 3 shows the association between individual sleep behavior and the risk of Scoliosis, adjusting for all covariates age, sex, ethnicity, household income, educational level Townsend deprivation index, body mass index, smoking status, alcohol status, diabetes, hypertension and after MET time per week usually experiencing insomnia and having excessive daytime sleepiness were associated with an increased risk of Scoliosis. Except for having a sleep duration of 7–8 h per day and having an evening chronotype. We report an unexpected finding: snoring serves as a protective factor against scoliosis. In our subgroup analysis, we categorized BMI into tertiles and observed that this protective effect was absent in both Tertile 1 and Tertile 2. Notably, snoring emerged as a protective factor for scoliosis solely within the Tertile 3 group and among individuals classified as overweight (BMI > 30 kg/m^2^) (Supplementary Table 4) (Yuan et al., 2022). Furthermore, in both the overall and overweight populations, the BMI of individuals who snored was significantly higher than that of non-snorers (*P* < 0.001) (Supplementary Table 5).

### 3.3 Subgroup analyses

We conducted subgroup analyses based on sex, age, educational level, diabetes, hypertension, and MET time per week to identify potential regulatory influencing factors. However, no significant interactions were observed except for diabetes. We found that for participants without diabetes, there was a significant negative correlation between sleep scores and the risk of scoliosis, but this correlation did not exist in participants with diabetes (*P* interaction < 0.05) (Table 3). A comparison of a healthy sleep score of 5 with a sleep score of 0–1 showed that the HR (95% CI) for scoliosis was 0.61 (0.49, 0.76) in individuals without diabetes and 1.65 (0.71, 3.81) in individuals with diabetes (Table 3).

**TABLE 3:** Associations between healthy sleep score and the risk of incident scoliosis stratified by subgroups.

Subgroups	Adjusted hazard ratio* (95%CI)					*P* for trend	*P* for interaction
	Healthy sleep score						
	0–1	2	3	4	5		
Age							0.087
< 60 Years (*n* = 232,823)	1 (Reference)	1.07 (0.74, 1.56)	0.89 (0.62, 1.27)	0.78 (0.54, 1.12)	0.70 (0.48, 1.02)	<0.001	
≥ 60 Years (*n* = 176,047)	1 (Reference)	0.78 (0.60, 1.03)	0.75 (0.58, 0.96)	0.67 (0.52, 0.86)	0.63 (0.48, 0.82)	< 0.001	
Sex							0.596
Male (*n* = 184,117)	1 (Reference)	1.01 (0.67, 1.54)	0.70 (0.46, 1.04)	0.71 (0.48, 1.06)	0.74 (0.48, 1.12)	0.019	
Female (*n* = 224,753)	1 (Reference)	0.81 (0.63, 1.05)	0.81 (0.63, 1.03)	0.69 (0.54, 0.88)	0.62 (0.48, 0.80)	< 0.001	
Education							0.610
College/University (*n* = 134,633)	1 (Reference)	0.97 (0.60, 1.56)	0.73 (0.46, 1.16)	0.74 (0.47, 1.17)	0.62 (0.39, 0.98)	< 0.001	
Other (*n* = 274,237)	1 (Reference)	0.83 (0.65, 1.07)	0.79 (0.63, 1.00)	0.68 (0.54, 0.86)	0.66 (0.51, 0.84)	< 0.001	
Household income							0.843
< £52,000 (*n* = 302,575)	1 (Reference)	0.84 (0.67, 1.06)	0.76 (0.61, 0.95)	0.68 (0.54, 0.84)	0.63 (0.50, 0.79)	<0.001	
≥ £52,000 (*n* = 106,295)	1 (Reference)	1.01 (0.53, 1.93)	0.88 (0.48, 1.63)	0.79 (0.43, 1.46)	0.72 (0.38, 1.34)	0.022	
Activity							0.703
< 40 (*n* = 249,898)	1 (Reference)	0.81 (0.62, 1.05)	0.74 (0.58, 0.95)	0.65 (0.51, 0.84)	0.65 (0.51, 0.85)	<0.001	
≥ 40 (*n* = 158,972)	1 (Reference)	1.00 (0.67, 1.51)	0.89 (0.60, 1.30)	0.81 (0.55, 1.19)	0.66 (0.45, 0.98)	< 0.001	
Diabetes							0.043
No (*n* = 387,930)	1 (Reference)	0.84 (0.67, 1.05)	0.75 (0.60, 0.93)	0.67 (0.54, 0.83)	0.61 (0.49, 0.76)	< 0.001	
Yes (*n* = 20,940)	1 (Reference)	1.15 (0.50, 2.69)	1.26 (0.57, 2.80)	1.07 (0.48, 2.41)	1.65 (0.71, 3.81)	0.323	
Hypertension							0.945
No (*n* = 299,501)	1 (Reference)	0.93 (0.68, 1.26)	0.78 (0.58, 1.04)	0.70 (0.52, 0.93)	0.66 (0.49, 0.89)	< 0.001	
Yes (*n* = 109,369)	1 (Reference)	0.79 (0.57, 1.08)	0.79 (0.58, 1.06)	0.71 (0.53, 0.96)	0.62 (0.45, 0.86)	0.001	

* Hazard ratios were adjusted for age, sex, ethnicity, household income, educational level, Townsend deprivation index, body mass index, smoking status, alcohol status, diabetes, hypertension, and Total weekly activity time. BMI, body mass index; CI, confidence interval.

**FIGURE 3:**
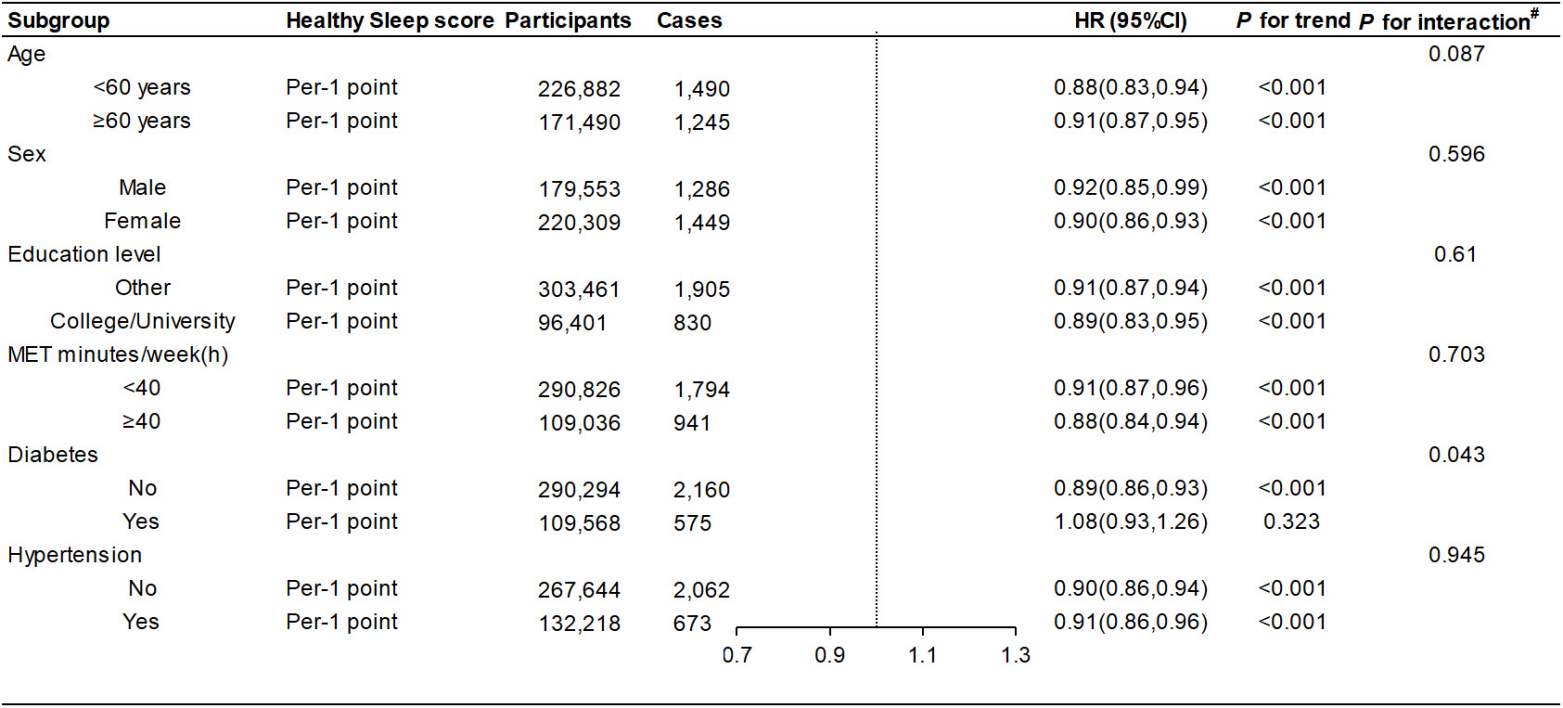
Subgroup analysis for the association between per-1 point increment of healthy sleep score and incident scoliosis. BMI (kg/m^2^): body mass index.

Subgroup analyses were performed based on sex, age, educational level, diabetes, hypertension, and MET time per week. The association between higher healthy sleep scores and a reduced risk of scoliosis was consistently observed (Supplementary Tables 6–11). Furthermore, the *P*-values for gender, age, educational level, diabetes, hypertension, and MET time per week across all subgroups were < 0.05. Similarly, a 1-point increase in the healthy sleep score yielded consistent results in the subgroup analyses for sex, age, educational level, diabetes, hypertension, and MET time per week, with all *P-*values remaining below 0.05 (Figure 3 and Supplementary Tables 6–11).

### 3.4 Sensitivity analysis

In sensitivity analyses, the association between healthy sleep scores and scoliosis risk remained robust. First, after excluding individuals who developed scoliosis within 1, 2, 5, or 10 years of follow-up, we observed similar associations. Second, we retained only white participants, excluding individuals from other racial groups. Third, we excluded participants who were enrolled after the outbreak. The results obtained from these analyses were consistent. Fourth, we constructed a weighted healthy sleep score based on five sleep behaviors, with the weights derived from the adjusted hazard ratios (HRs) obtained from a Cox proportional hazards model (Supplementary Table 12). Fifth, we performed multiple imputation on the missing sleep data and repeated the primary analysis, yielding stable results (Supplementary Table 13). Finally, sensitivity analysis using the Fine-Gray competing risk model yielded results consistent with the primary Cox model (Supplementary Table 14).

## 4 Discussion

Our primary results indicate that healthier sleep patterns exhibit a dose-response relationship with a significantly reduced risk of developing scoliosis. Specifically, for each 1-point increase in the healthy sleep score, the risk of new-onset scoliosis decreases by 10%. In comparison to individuals with the poorest sleep patterns (0–1 healthy behaviors), participants exhibiting all five healthy sleep behaviors demonstrated a 36% lower risk of developing the condition. Notably, we identified a significant interaction: this protective association was pronounced in the non-diabetic population, whereas no statistical significance was found among diabetic patients. These findings offer a new perspective on the etiology of scoliosis and underscore the importance of sleep health as a potential public health intervention target for preserving spinal structural integrity.

The findings of this study address a significant knowledge gap in the existing literature. Prior investigations into the relationship between sleep and scoliosis have predominantly utilized cross-sectional designs, focusing primarily on diagnosed adolescent or adult patients with scoliosis (Van Looveren et al., 2021; Yakut et al., 2022). For instance, previous studies have shown that patients with idiopathic scoliosis usually have poor sleep quality, which is associated with pain and depressive symptoms (Ugur et al., 2023). The inherent limitation of these studies is their inability to ascertain the causal direction. Our study, employing a prospective design, excluded individuals with scoliosis at baseline, thereby demonstrating that exposure to sleep pattern variations precedes disease outcomes over time. This approach significantly bolsters the evidence supporting the hypothesis that sleep patterns may act as an independent risk factor for scoliosis, representing the most significant contribution of this study to the existing literature.

Although directly comparable prospective studies are lacking, our findings align with broader research on sleep and musculoskeletal health. Numerous studies have demonstrated that both insufficient and excessive sleep duration, as well as poor sleep quality, are linked to an increased risk of osteoporosis, decreased bone density, and heightened fracture risk (Ochs-Balcom et al., 2020; Tian et al., 2024). Additionally, sleep disturbances have been identified as significant predictors of the onset and persistence of chronic back pain (Zhong and Wang, 2024). The pathological basis of adult scoliosis frequently includes osteoporosis, intervertebral disc degeneration, and paravertebral muscle dysfunction. Consequently, our research findings can be viewed as an extension and expansion of the existing evidence regarding new-onset spinal deformities. These results indicate that a healthy sleep pattern may serve as a significant protective factor in maintaining overall spinal health homeostasis.

Previous studies have shown that a healthy sleep rhythm, especially a morning preference for going to bed early and getting up early and a regular schedule, ensures that melatonin is secreted at a normal rhythm and peak in the dark environment at night. Melatonin is not only an important hormone for sleep, but also a hormone that has a direct anabolic effect on bones (Ahuja et al., 2018). Long-term poor sleep habits can disrupt the physiological rhythm of melatonin, resulting in a decrease in its overall secretion. This disruption may disturb the balance between bone formation and bone resorption, thereby rendering bones more fragile and increasing the risk of spinal deformation under stress loads (Cai et al., 2021; Souissi and Dergaa, 2022). Adequate sleep duration and high-quality deep sleep represent a critical period for the secretion of growth hormone (GH). GH plays an equally vital role in adults, as it helps maintain bone mass, promotes protein synthesis, and preserves muscle mass and strength by stimulating the liver to produce insulin-like growth factor-1 (IGF-1) (Acaroglu et al., 2012). The paravertebral core muscle group serves as the framework that upholds the dynamic stability of the spine. Prolonged sleep deprivation can impair GH secretion, potentially resulting in atrophy and a decline in the strength of the paravertebral muscles. This deterioration diminishes their capacity to counteract gravity and asymmetric loads, thereby increasing the spine’s susceptibility to instability and scoliosis. Additionally, sleep disorders, including insomnia and frequent nocturnal awakenings, can activate the body’s stress response, leading to elevated cortisol levels throughout the day, particularly the non-physiological secretion of cortisol during the night (Jurado-Fasoli et al., 2018). Chronic hypercortisolemia exerts profound catabolic effects on the musculoskeletal system, notably by inhibiting osteoblast function, increasing bone calcium loss, and accelerating muscle protein breakdown (Osella et al., 2012). This persistent state of degradation directly compromises the bones and muscles that constitute the supportive framework of the spine, thereby fostering pathophysiological conditions conducive to the onset and progression of degenerative scoliosis (Jurado-Fasoli et al., 2018). While experimental data support melatonin’s osteogenic potential, direct evidence linking circadian disruption to altered melatonin secretion and subsequent scoliosis risk in humans remains limited. The observed association may therefore reflect broader sleep-health optimization rather than melatonin-specific pathways.

Sleep is a critical period for immune regulation and memory consolidation. Chronic sleep deprivation or poor sleep quality induces systemic inflammation, increasing proinflammatory cytokines such as tumor necrosis factor-α (TNF-α), interleukin-1 (IL-1), and interleukin-6 (IL-6) (Cai et al., 2021; Jurado-Fasoli et al., 2018). These mediators contribute to spinal degeneration by degrading intervertebral disc matrix, triggering chondrocyte apoptosis, and promoting osteophyte formation in small joints (Grandner et al., 2013; Irwin, 2015). Maintaining healthy sleep suppresses this systemic inflammatory response and may delay or prevent the degenerative processes that lead to adult degenerative scoliosis (Ditmer et al., 2021).

Beyond hormonal and inflammatory pathways, musculoskeletal and biomechanical mechanisms may also link sleep to scoliosis onset. Sleep duration and quality influence muscle recovery and repair. Short or fragmented sleep can impair the function of paraspinal muscles and hinder their recovery after daytime loading, potentially weakening the muscular support that preserves spinal alignment. Sleep disorders also alter pain perception and reduce physical capacity, which can promote compensatory postures and asymmetric loading that aggravate spinal curvature (Runge et al., 2024). Future studies should use objective measures of sleep posture and nocturnal spinal loading to elucidate these biomechanical pathways.

Sleep, especially rapid eye movement (REM) sleep and deep sleep, is crucial for the functional recovery of the central nervous system, the consolidation of learning and memory, and the improvement of motor skills (Brawn et al., 2008). Daytime sleepiness is a direct consequence of insufficient sleep, which can reduce alertness, reaction time and fine motor control ability (Vargas et al., 2020). This may affect an individual’s ability to maintain the correct posture during daily activities, causing the spine to be in an asymmetrical stress state for a long time. In addition, sleep is a crucial period for the repair of micro-damage to muscle tissue and the replenishment of energy reserves (Cai et al., 2021). Healthy sleep allows paravertebral muscle groups to recover fully after daytime activity, restoring their tension and endurance and resisting cumulative stress that can lead to spinal deformity. We observed an unexpected finding: snoring appeared as a protective factor against scoliosis. The apparent protective association between snoring and scoliosis risk was limited to individuals with higher adiposity (BMI > 30), as shown in our subgroup analysis. This pattern indicates effect modification by BMI, implying that the influence of snoring on spinal health differs across adiposity levels. In participants with high BMI, snoring may reflect underlying metabolic or respiratory conditions that affect bone health differently than in normal-weight individuals (Campos et al., 2020). The absence of an association in lower-BMI groups may also result from limited statistical power due to fewer events in those subgroups. Finally, although we adjusted for BMI, residual confounding may persist and vary across BMI strata (Campos et al., 2020; Feigin et al., 2025), which could influence the observed relationship.

Our results indicate that among participants with a sleep score of 5, the PAR% for scoliosis is 8.53%. The reported PAR% of 8.53% represents a theoretical maximum under strong assumptions of causality, behavioral stability, and independent modifiability of all five sleep components. In practice, population-wide achievement of perfect sleep health is unlikely, and the true preventable fraction under realistic intervention scenarios would be substantially lower. Furthermore, unmeasured confounding and the single baseline assessment of sleep patterns may bias this estimate in either direction. Additionally, the exclusion of 91,705 participants (18.3%) due to missing sleep data raises concerns regarding potential selection bias. Consistent with previous findings in the UKB cohort (Swanson, 2012), individuals with missing data were generally older, socioeconomically disadvantaged, and exhibited poorer health profiles. This systematic exclusion may result in an underestimation of the true incidence of scoliosis and could attenuate observed associations toward the null. Nevertheless, sensitivity analyses employing multiple imputation for missing sleep variables produced effect estimates that aligned with those from the complete-case analysis. This suggests that while selection bias may impact generalizability, it does not fundamentally alter the direction or significance of the observed relationships.

Another novel and important finding of this study is that the protective effect of healthy sleep on scoliosis is absent in patients with diabetes. This result implies that diabetes may fundamentally alter the relationship between sleep and spinal health. A likely explanation is that diabetes, particularly type 2 diabetes, is a metabolic disorder that adversely affects the musculoskeletal system. Chronic hyperglycemia promotes accumulation of advanced glycation end products in bone collagen and the intervertebral disc matrix, which increases tissue stiffness and fragility, compromises biomechanical integrity, and triggers local inflammation and oxidative stress (Yang et al., 2022). We acknowledge that the interaction term (*P* = 0.043) reaches statistical significance but warrants cautious interpretation. The diabetes subgroup comprised only 20,940 participants (5.1% of the total), and the scoliosis incidence in this subgroup was approximately 0.73%, yielding roughly 153 events. With so few events, the effect estimate is highly imprecise (HR 1.65, 95% CI 0.71–3.81). Thus, the apparent significant interaction likely reflects the pronounced protective effects observed in the non-diabetic subgroups rather than true biological differences within the diabetic subgroup. At the same time, improving sleep remains crucial for blood sugar control and overall health of diabetic patients. Despite these limitations, we maintain that improving sleep is important for glycemic control in patients with diabetes and for the overall health status of individuals with scoliosis. This hypothesis can be tested in a larger diabetes cohort in future studies.

## 5 Strengths and limitations

This study adopted a prospective cohort design and an extremely large sample size, providing strong statistical power and a reliable causal inference basis for the research results. Secondly, a follow-up period of nearly 16 years is sufficient to capture the occurrence process of a relatively slow-developing chronic disease like adult scoliosis. Thirdly, we have adopted a multi-dimensional and comprehensive sleep score instead of relying on a single sleep indicator. This can more comprehensively reflect an individual’s overall sleep health status and is in line with the current research trends in the field of sleep medicine (Pu et al., 2024). Various sensitivity analyses have also yielded reliable findings. This study has several limitations. First, the assessment of sleep behavior is based on self-reporting, which may introduce recall bias and measurement errors. Second, the participants in the UKB cohort predominantly belong to European white ancestry, potentially limiting the generalizability of the findings to other racial or broader populations (Quintero Santofimio et al., 2023). Third, sleep patterns and other lifestyle factors were evaluated only once at baseline. Over the follow-up period exceeding 10 years, these behaviors may have changed, and such dynamic shifts could introduce bias in risk assessment. Fourth, our outcome (ICD-10 M41) groups etiologically distinct scoliosis subtypes. Although we excluded congenital and perinatal cases by removing diagnoses with event dates near birth, we could not distinguish degenerative scoliosis from long-standing idiopathic scoliosis. Future studies should address this limitation using clinical or radiographic data. Fifth, considerations regarding potential reverse causal relationships in observational studies. However, the prospective design and the sensitivity analysis that excluded cases diagnosed within the 5–10 years prior to the follow-up (with consistent results) alleviated this concern. Nevertheless, residual reverse causal relationships cannot be completely ruled out. Further research is needed in the future to determine the direction of the causal relationship. Sixth, We acknowledge that ICD-10 code M41 denotes a broad category of spinal curvature encompassing multiple subtypes and etiologies. Therefore, our findings should be interpreted as associations with mixed spinal curvatures of diverse causes rather than as specific associations with degenerative spinal curvature. Future studies that analyze subtype-specific data will be necessary to determine whether these associations apply to each subtype. Finally, UKB participants are generally healthier than the broader British population, which may bias results toward underestimating the true incidence of scoliosis and the prevalence of severe sleep deprivation patterns in the general population. Future studies should employ more representative population samples to assess the generalizability of these findings.

## 6 Conclusion

In conclusion, this large-scale prospective study utilizing data from the UKB provides the most robust evidence to date that adherence to a multifaceted healthy sleep pattern is significantly linked to a decreased risk of new-onset scoliosis in middle-aged and older adults. This protective association is particularly pronounced in non-diabetic populations. These findings not only pave the way for new research into the intricate etiology of scoliosis but also underscore the significance of sleep health as a modifiable and promising public health strategy for preserving spinal structural integrity and preventing associated deformities. Future research should aim to validate these findings and investigate the underlying biological mechanisms involved.

## Data Availability

The raw data supporting the conclusions of this article will be made available by the authors, without undue reservation.
