# Supplementary material for: The relationship between healthy sleep patterns and the risk of scoliosis: a large prospective cohort study
Source: Front Neurosci. 2026 Jun 17;20:1839503. doi: 10.3389/fnins.2026.1839503 (PMC13320351; doi:10.3389/fnins.2026.1839503)
Supplement: Supplementary file 1 [file Data_Sheet_1.docx]

SUPPLEMENTARY MATERIALS

**The relationship between healthy sleep patterns and the risk of scoliosis: A Large Prospective Cohort Study**

Wanyue Li^1,2†^, Shuxiao Ma^1†^, Mei Hu^1†^, Yanzhao Hao^1^, Ying Li^2^, Xiguo Cai^2^, Weisheng Zhuang^2*^ and Ruihua Sun^1*^

**Supplementary Table 1. The Assessment of sleep behaviors**

**Supplementary Table 2. Multivariable-adjusted HRs (95% CIs) for scoliosis by sleep behaviours**

**Supplementary Table 3. Risk of scoliosis associated with snoring stratified by BMI**

**Supplementary Table 4. To compare the differences in BMI between the snoring and non-snoring groups**

**in all participants and overweight participants, respectively**

**Supplementary Table 5. Risk of scoliosis associated with healthy sleep score stratified by sex**

**Supplementary Table 6. Risk of scoliosis associated with healthy sleep score stratified by age**

**Supplementary Table 7. Risk of scoliosis associated with healthy sleep score stratified by educational level**

**Supplementary Table 8. Risk of scoliosis associated with healthy sleep score stratified by MET time per week**

**Supplementary Table 9. Risk of scoliosis associated with healthy sleep score stratified by diabetes**

**Supplementary Table 10. Risk of scoliosis associated with healthy sleep score stratified by hypertension**

**Supplementary Table 11. Sensitivity analyses regarding risk of incident scoliosis according to healthy sleep score**

**Supplementary Table 1. The Assessment of sleep behaviors**

| **Characteristics** | **UK Biobank Code** | **UK Biobank Questionnaire** | **Healthy sleep(%)** | **Unhealthy sleep (%)** |
| --- | --- | --- | --- | --- |
| **Sleep duration** | 1160 | About how many hours sleep do you get in every 24 hours? (please include naps) | 7-8 h/d | <7 or ≥9 h/d. |
| **Chronotype** | 1180 | Do you consider yourself to be? | Definitely a "morning" person; More a "morning" than "evening" person | More an "evening" than a "morning person; Definitely an "evening" person. |
| **Daytime sleepiness** | 1220 | How likely are you to doze off or fall asleep during the daytime when you don't mean to? (e.g. when working. reading or driving) | Never/rarely/Sometimes | Often/All the Time |
| **Insomnia** | 1200 | Do you have trouble falling asleep at night or do you wake up in the middle of the night? | Never/rarely/Sometimes | Usually |
| **Snoring** | 1210 | Does your partner or a close relative or friend complain about your snoring? | No | Yes |

**Supplementary Table 2. Comparison of baseline characteristics between excluded and included subjects**

| **Characteristics** | **Overall** | **Group** | | ***P* -value** |
| --- | --- | --- | --- | --- |
|  |  | **Inclusion group** | **Exclusion group** |  |
|  | **(N=501936)** | **(N=410231)** | **(N=91705)** |  |
| Age | 56.5 (8.09) | 56.5 (8.09) | 56.8 (8.10) | <0.001 |
| Townsend deprivation index | -1.29 (3.10) | -1.41 (3.03) | -0.792 (3.32) | <0.001 |
| Diabetes Yes | 26618 (5.3%) | 21007 (5.1%) | 5611 (6.1%) | <0.001 |
| No | 475318 (94.7%) | 389224 (94.9%) | 86094 (93.9%) |  |
| Hypertension Yes | 209310 (41.7%) | 169228 (41.3%) | 40082 (43.7%) | <0.001 |
| No | 292626 (58.3%) | 241003 (58.7%) | 51623 (56.3%) | <0.001 |

The values for continuous variables are given as mean±SD and values for categorical variables are given as numbers (percentage).

**Supplementary Table 3. Multivariable-adjusted HRs (95% CIs) for scoliosis by sleep behaviours**

|  | | | | | | | | | | |
| --- | --- | --- | --- | --- | --- | --- | --- | --- | --- | --- |
| **Sleep behaviours** | **Participants** | **Cases** | **Model 1** | |  | **Model 2** | |  | **Model 3** | |
|  |  |  | **HRs (95%CIs)** | ***P*** |  | **HRs (95%CIs)** | ***P*** |  | **HRs (95%CIs)** | ***P*** |
| Sleep duration |  |  |  |  |  |  |  |  |  |  |
| Normal (7-9 h) | 302709 | 2034 | 1 (reference) | - |  | 1 (reference) | - |  | 1 (reference) | - |
| Short (<7 h) | 98764 | 870 | 1.32(1.22,1.43) | <0.001 |  | 1.29(1.20,1.40) | <0.001 |  | 1.28(1.18,1.38) | <0.001 |
| Long (>9 h) | 7397 | 72 | 1.56(1.23,1.97) | <0.001 |  | 1.29(1.02,1.63) | 0.037 |  | 1.25(0.98,1.58) | 0.067 |
| Chronotype |  |  |  |  |  |  |  |  |  |  |
| Morningness | 110807 | 870 | 1 (reference) | - |  | 1 (reference) | - |  | 1 (reference) | - |
| Morningness than eveningness | 145748 | 1020 | 0.89(0.81,0.97) | 0.008 |  | 0.96(0.88,1.05) | 0.365 |  | 0.97(0.88,1.06) | 0.471 |
| Eveningness than morningness | 116241 | 827 | 0.90(0.82,0.99) | 0.037 |  | 1.03(0.94,1.13) | 0.561 |  | 1.02(0.93,1.13) | 0.638 |
| Eveningness | 36074 | 259 | 0.92(0.80,1.05) | 0.223 |  | 1.11(0.96,1.27) | 0.159 |  | 1.08(0.94,1.24) | 0.293 |
| Daytime sleepiness |  |  |  |  |  |  |  |  |  |  |
| Never/rarely | 311365 | 2080 | 1 (reference) | - |  | 1 (reference) | - |  | 1 (reference) | - |
| Sometimes | 86296 | 764 | 1.35(1.24,1.47) | <0.001 |  | 1.16(1.07,1.26) | <0.001 |  | 1.16(1.07,1.26) | <0.001 |
| Always/Often | 11209 | 132 | 1.83(1.54,2.19) | <0.001 |  | 1.64(1.37,1.95) | <0.001 |  | 1.62(1.35,1.93) | <0.001 |
| Insomnia |  |  |  |  |  |  |  |  |  |  |
| Never/rarely | 99704 | 511 | 1 (reference) | - |  | 1 (reference) | - |  | 1 (reference) | - |
| Sometimes | 195565 | 1340 | 1.34(1.21,1.49) | <0.001 |  | 1.05(0.95,1.17) | 0.339 |  | 1.05(0.94,1.16) | 0.386 |
| Usually | 113601 | 1125 | 1.96(1.77,2.18) | <0.001 |  | 1.39(1.25,1.55) | <0.001 |  | 1.37(1.23,1.52) | <0.001 |
| Snoring |  |  |  |  |  |  |  |  |  |  |
| No | 256813 | 2055 | 1 (reference) | - |  | 1 (reference) | - |  | 1 (reference) | - |
| Yes | 152057 | 921 | 0.76(0.70,0.82) | <0.001 |  | 0.88(0.82,0.96) | 0.002 |  | 0.87(0.81,0.95) | <0.001 |

A Cox proportional hazard model was conducted. Model 1: unadjusted  model; Model 2, age, sex, ethnicity, household income,educational level, and Townsend deprivation index were adjusted for in this model; Model 3, body mass index, smoking status, alcohol status, diabetes, hypertension, and MET time per week additionally were adjusted for in this model.

HR, hazard ratio; CI, confidence interval.

**Supplementary Table 4. Risk of scoliosis associated with snoring stratified by BMI**

| **BMI subgroup** | **Snoring** | | ***P*** | ***P* for interaction** |
| --- | --- | --- | --- | --- |
|  | **Yes** | **No** |  |  |
| **Tertile 1 (N=102213)** |  |  |  | 0.108 |
| Participants, n | 22827 | 79386 | **-** |  |
| Incident scoliosis, n | 167 | 629 | **-** |  |
| Adjusted HR (95% CI) | |  |  |  |
| Adjusted model | Reference | 1.04(0.87,1.24) | 0.666 |  |
| **Tertile 2 (N=204554)** |  |  |  |  |
| Participants, n | 77787 | 126767 | - |  |
| Incident scoliosis, n | 458 | 968 | - |  |
| Adjusted HR (95% CI) | |  |  |  |
| Adjusted model | Reference | 1.09(0.98,1.22) | 0.127 |  |
| **Tertile 3 (N=102103)** |  |  |  |  |
| Participants, n | 51443 | 50660 | - |  |
| Incident scoliosis, n | 296 | 458 | - |  |
| Adjusted HR (95% CI) |  |  |  |  |
| Adjusted model | Reference | 1.32(1.14,1.53) | <0.001 |  |

A Cox proportional hazard model was conducted. Adjusted model , age,sex, ethnicity, educational level, Townsend deprivation index, body mass index, smoking status, alcohol status, diabetes, hypertension, and MET time per week were adjusted for in this model

CI, confidence interval. BMI: body mass index (kg/m^2^)

**Supplementary Table 5. To compare the differences in BMI between the snoring and non-snoring groups in all participants and overweight participants, respectively**

| **t-test** | **Snoring** | | ***P*** |
| --- | --- | --- | --- |
|  | **Yes** | No |  |
| **BMI (N=408870)** |  |  |  |
| Participants, n | 152057 | 256813 | **-** |
| Incident scoliosis, n | 921 | 2055 | **-** |
| mean±SD | 28.69 (4.88) | 26.64 (4.54) | <0.001 |
| **BMI > 30 kg/m^2^ (N=99034)** |  |  |  |
| Participants, n | 50016 | 49018 | - |
| Incident scoliosis, n | 291 | 444 | - |
| mean±SD | 34.11 (3.98) | 33.73 (3.74) | <0.001 |

BMI: body mass index

**Supplementary Table 6. Risk of scoliosis associated with healthy sleep score stratified by sex**

| **Sex subgroup** | **Healthy sleep score** | | | | | **Per 1-point increment** | ***P* for trend** |
| --- | --- | --- | --- | --- | --- | --- | --- |
|  | **0-1** | **2** | **3** | **4** | **5** |  |  |
| **Male (N=184117)** |  |  |  |  |  |  |  |
| Participants, n | 4872 | 21933 | 55701 | 69233 | 32378 | - | - |
| Incident scoliosis, n | 27 | 120 | 209 | 266 | 128 | - | - |
| Hazard ratio for incident scoliosis (95% CI) | |  |  |  |  |  |  |
| Crude model | Reference | 0.98(0.64,1.48) | 0.66(0.44,0.99) | 0.67(0.45,1.00) | 0.69(0.46,1.05) | 0.91(0.85,0.97) | 0.007 |
| Adjusted model 1 | Reference | 1.00(0.66,1.52) | 0.68(0.46,1.02) | 0.70(0.47,1.03) | 0.71(0.47,1.08) | 0.91(0.85,0.98) | 0.010 |
| Adjusted model 2 | Reference | 1.01(0.67,1.54) | 0.70(0.46,1.04) | 0.71(0.48,1.06) | 0.74(0.48,1.12) | 0.92(0.85,0.99) | 0.019 |
| **Female (N=224753)** |  |  |  |  |  |  |  |
| Participants, n | 5067 | 24242 | 59393 | 81054 | 54997 | - | - |
| Incident scoliosis, n | 73 | 283 | 676 | 746 | 448 | - | - |
| Hazard ratio for incident scoliosis (95% CI) | |  |  |  |  |  |  |
| Crude model | Reference | 0.80(0.62,1.03) | 0.78(0.61,0.99) | 0.62(0.49,0.79) | 0.55(0.43,0.70) | 0.86(0.83,0.90) | <0.001 |
| Adjusted model 1 | Reference | 0.81(0.62,1.04) | 0.79(0.62,1.01) | 0.67(0.53,0.85) | 0.60(0.47,0.77) | 0.89(0.85,0.93) | <0.001 |
| Adjusted model 2 | Reference | 0.81(0.63,1.05) | 0.81(0.63,1.03) | 0.69(0.54,0.88) | 0.62(0.48,0.80) | 0.90(0.86,0.93) | <0.001 |

A Cox proportional hazard model was conducted. Adjusted model 1, age, ethnicity, household income,educational level, and Townsend deprivation index were adjusted for in this model; adjusted model 2, body mass index, smoking status, alcohol status, diabetes, hypertension, and MET time per week additionally were adjusted for in this model.

CI, confidence interval.

**Supplementary Table 7. Risk of scoliosis associated with healthy sleep score stratified by age**

| **Age subgroup** | **Healthy sleep score** | | | | | **Per 1-point increment** | ***P* for trend** |
| --- | --- | --- | --- | --- | --- | --- | --- |
|  | **0-1** | **2** | **3** | **4** | **5** |  |  |
| **<60 y (N=232823)** |  |  |  |  |  |  |  |
| Participants, n | 5785 | 26222 | 64644 | 85740 | 50432 | - | - |
| Incident scoliosis, n | 34 | 151 | 289 | 329 | 181 | - | - |
| Hazard ratio for incident scoliosis (95% CI) | |  |  |  |  |  |  |
| Crude model | Reference | 0.97(0.67,1.40) | 0.75(0.52,1.07) | 0.64(0.45,0.91) | 0.59(0.41,0.86) | 0.86(0.81,0.91) | <0.001 |
| Adjusted model 1 | Reference | 1.01(0.70,1.47) | 0.81(0.57,1.16) | 0.69(0.49,0.99) | 0.61(0.42,0.88) | 0.86(0.81,0.91) | <0.001 |
| Adjusted model 2 | Reference | 1.07(0.74,1.56) | 0.89(0.62,1.27) | 0.78(0.54,1.12) | 0.70(0.48,1.02) | 0.88(0.83,0.94) | 0.008 |
| **≥60 y (N=176047)** |  |  |  |  |  |  |  |
| Participants, n | 4154 | 19953 | 50450 | 64547 | 36943 | - | - |
| Incident scoliosis, n | 66 | 252 | 596 | 683 | 395 | - | - |
| Hazard ratio for incident scoliosis (95% CI) | |  |  |  |  |  |  |
| Crude model | Reference | 0.78(0.60,1.03) | 0.72(0.56,0.93) | 0.64(0.50,0.83) | 0.64(0.50,0.84) | 0.92(0.88,0.96) | <0.001 |
| Adjusted model 1 | Reference | 0.78(0.60,1.03) | 0.72(0.56,0.93) | 0.64(0.50,0.83) | 0.64(0.50,0.84) | 0.91(0.87,0.95) | <0.001 |
| Adjusted model 2 | Reference | 0.78(0.60,1.03) | 0.75(0.58,0.96) | 0.67(0.52,0.86) | 0.63(0.48,0.82) | 0.91(0.87,0.95) | <0.001 |

A Cox proportional hazard model was conducted. Adjusted model 1, sex, ethnicity, household income,educational level, and Townsend deprivation index were adjusted for in this model; adjusted model 2, body mass index, smoking status, alcohol status, diabetes, hypertension, and MET time per week additionally were adjusted for in this model.

CI, confidence interval.

**Supplementary Table 8. Risk of scoliosis associated with healthy sleep score stratified by educational level**

| **Education subgroup** | **Healthy sleep score** | | | | | **Per 1-point increment** | ***P* for trend** |
| --- | --- | --- | --- | --- | --- | --- | --- |
|  | **0-1** | **2** | **3** | **4** | **5** |  |  |
| **College/University (N=134633)** |  |  |  |  |  |  |  |
| Participants, n | 2433 | 12681 | 35631 | 51809 | 32079 | - | - |
| Incident scoliosis, n | 20 | 103 | 211 | 307 | 164 | - | - |
| Hazard ratio for incident scoliosis (95% CI) | |  |  |  |  |  |  |
| Crude model | Reference | 0.98(0.61,1.58) | 0.71(0.45,1.12) | 0.71(0.45,1.11) | 0.61(0.38,0.96) | 0.88(0.82,0.94) | <0.001 |
| Adjusted model 1 | Reference | 0.96(0.60,1.56) | 0.72(0.46,1.14) | 0.72(0.46,1.14) | 0.60(0.38,0.95) | 0.88(0.82,0.94) | <0.001 |
| Adjusted model 2 | Reference | 0.97(0.60,1.56) | 0.73(0.46,1.16) | 0.74(0.47,1.17) | 0.62(0.39,0.98) | 0.89(0.83,0.95) | <0.001 |
| **Other (N=274237)** |  |  |  |  |  |  |  |
| Participants, n | 7506 | 33494 | 79463 | 98478 | 55296 | - | - |
| Incident scoliosis, n | 80 | 300 | 674 | 705 | 412 | - | - |
| Hazard ratio for incident scoliosis (95% CI) | |  |  |  |  |  |  |
| Crude model | Reference | 0.83(0.65,1.06) | 0.78(0.62,0.98) | 0.65(0.52,0.82) | 0.68(0.53,0.86) | 0.91(0.87,0.95) | <0.001 |
| Adjusted model 1 | Reference | 0.82(0.64,1.05) | 0.78(0.62,0.98) | 0.66(0.52,0.83) | 0.64(0.50,0.81) | 0.90(0.86,0.94) | <0.001 |
| Adjusted model 2 | Reference | 0.83(0.65,1.07) | 0.79(0.63,1.00) | 0.68(0.54,0.86) | 0.66(0.51,0.84) | 0.91(0.87,0.94) | <0.001 |

A Cox proportional hazard model was conducted. Adjusted model 1, age,sex, ethnicity, household income, and Townsend deprivation index were adjusted for in this model; adjusted model 2, body mass index, smoking status, alcohol status, diabetes, hypertension, and MET time per week additionally were adjusted for in this model.

CI, confidence interval.

**Supplementary Table 9. Risk of scoliosis associated with healthy sleep score stratified by MET time per week**

| **Townsend deprivation index subgroup** | **Healthy sleep score** | | | | | **Per 1-point increment** | ***P* for trend** |
| --- | --- | --- | --- | --- | --- | --- | --- |
|  | **0-1** | **2** | **3** | **4** | **5** |  |  |
| **MET minutes/week(h)<40 (N=249898)** |  |  |  |  |  |  |  |
| Participants, n | 6640 | 29843 | 71874 | 90894 | 50647 | - | - |
| Incident scoliosis, n | 72 | 258 | 547 | 594 | 350 | - | - |
| Hazard ratio for incident scoliosis (95% CI) | |  |  |  |  |  |  |
| Crude model | Reference | 0.79(0.61,1.02) | 0.69(0.54,0.88) | 0.58(0.46,0.74) | 0.61(0.48,0.79) | 0.90(0.86,0.94) | <0.001 |
| Adjusted model 1 | Reference | 0.79(0.61,1.03) | 0.71(0.56,0.91) | 0.62(0.49,0.80) | 0.62(0.48,0.80) | 0.90(0.86,0.94) | <0.001 |
| Adjusted model 2 | Reference | 0.81(0.62,1.05) | 0.74(0.58,0.95) | 0.65(0.51,0.84) | 0.65(0.51,0.85) | 0.91(0.87,0.96) | <0.001 |
| **MET minutes/week(h) ≥40 (N=158972)** |  |  |  |  |  |  |  |
| Participants, n | 3299 | 16332 | 43220 | 59393 | 36728 | - | - |
| Incident scoliosis, n | 28 | 145 | 338 | 418 | 226 | - | - |
| Hazard ratio for incident scoliosis (95% CI) | |  |  |  |  |  |  |
| Crude model | Reference | 1.04(0.69,1.55) | 0.91(0.62,1.34) | 0.81(0.56,1.19) | 0.71(0.48,1.05) | 0.89(0.84,0.94) | <0.001 |
| Adjusted model 1 | Reference | 1.01(0.67,1.51) | 0.90(0.61,1.32) | 0.81(0.56,1.20) | 0.67(0.45,1.00) | 0.89(0.84,0.94) | <0.001 |
| Adjusted model 2 | Reference | 1.00(0.67,1.51) | 0.89(0.60,1.30) | 0.81(0.55,1.19) | 0.66(0.45,0.98) | 0.88(0.84,0.94) | <0.001 |

A Cox proportional hazard model was conducted. Adjusted model 1, age,sex, ethnicity, and household income,educational level were adjusted for in this model; adjusted model 2, body mass index, smoking status, alcohol status, diabetes, hypertension, and MET time per week additionally were adjusted for in this model.

CI, confidence interval.

**Supplementary Table 10. Risk of scoliosis associated with healthy sleep score stratified by diabetes**

| **Diabetes subgroup** | **Healthy sleep score** | | | | | **Per 1-point increment** | ***P* for trend** |
| --- | --- | --- | --- | --- | --- | --- | --- |
|  | **0-1** | **2** | **3** | **4** | **5** |  |  |
| **Diabetes No (N=387930)** |  |  |  |  |  |  |  |
| Participants, n | 8815 | 42676 | 108508 | 143540 | 84391 | - | - |
| Incident scoliosis, n | 93 | 379 | 836 | 971 | 547 | - | - |
| Hazard ratio for incident scoliosis (95% CI) | |  |  |  |  |  |  |
| Crude model | Reference | 0.83(0.66,1.04) | 0.72(0.58,0.89) | 0.63(0.51,0.78) | 0.60(0.48,0.75) | 0.89(0.86,0.92) | <0.001 |
| Adjusted model 1 | Reference | 0.83(0.66,1.04) | 0.73(0.59,0.91) | 0.65(0.53,0.80) | 0.58(0.47,0.73) | 0.89(0.85,0.92) | <0.001 |
| Adjusted model 2 | Reference | 0.84(0.67,1.05) | 0.75(0.60,0.93) | 0.67(0.54,0.83) | 0.61(0.49,0.76) | 0.89(0.86,0.93) | <0.001 |
| **Diabetes Yes (N=20940)** |  |  |  |  |  |  |  |
| Participants, n | 1124 | 3499 | 6586 | 6747 | 2984 | - | - |
| Incident scoliosis, n | 7 | 24 | 49 | 41 | 29 | - | - |
| Hazard ratio for incident scoliosis (95% CI) | |  |  |  |  |  |  |
| Crude model | Reference | 1.10(0.47,2.55) | 1.17(0.53,2.59) | 0.95(0.43,2.11) | 1.51(0.66,3.44) | 1.06(0.91,1.23) | 0.483 |
| Adjusted model 1 | Reference | 1.10(0.47,2.56) | 1.18(0.53,2.60) | 0.96(0.43,2.14) | 1.44(0.63,3.29) | 1.05(0.90,1.21) | 0.557 |
| Adjusted model 2 | Reference | 1.15(0.50,2.69) | 1.26(0.57,2.80) | 1.07(0.48,2.41) | 1.65(0.71,3.81) | 1.08(0.93,1.26) | 0.323 |

A Cox proportional hazard model was conducted. Adjusted model 1, age, sex, ethnicity, household income,educational level, and Townsend deprivation index were adjusted for in this model; adjusted model 2, smoking status, alcohol status, diabetes, hypertension, and MET time per week additionally were adjusted for in this model.

CI, confidence interval.

**Supplementary Table 11. Risk of scoliosis associated with healthy sleep score stratified by hypertension**

| **Hypertension subgroup** | **Healthy sleep score** | | | | | **Per 1-point increment** | ***P* for trend** |
| --- | --- | --- | --- | --- | --- | --- | --- |
|  | **0-1** | **2** | **3** | **4** | **5** |  |  |
| **Hypertension No (N=299501)** |  |  |  |  |  |  |  |
| Participants, n | 5929 | 30966 | 81467 | 112498 | 68641 | - | - |
| Incident scoliosis, n | 50 | 247 | 540 | 661 | 407 | - | - |
| Hazard ratio for incident scoliosis (95% CI) | | |  |  |  |  |  |
| Crude model | Reference | 0.94(0.69,1.27) | 0.77(0.58,1.03) | 0.68(0.51,0.91) | 0.69(0.51,0.92) | 0.90(0.86,0.94) | <0.001 |
| Adjusted model 1 | Reference | 0.94(0.69,1.27) | 0.79(0.59,1.06) | 0.71(0.53,0.95) | 0.68(0.50,0.91) | 0.90(0.86,0.94) | <0.001 |
| Adjusted model 2 | Reference | 0.93(0.68,1.26) | 0.78(0.58,1.04) | 0.70(0.52,0.93) | 0.66(0.49,0.89) | 0.90(0.86,0.94) | <0.001 |
| **Hypertension Yes (N=109369)** |  |  |  |  |  |  |  |
| Participants, n | 4010 | 15209 | 33627 | 37789 | 18734 | - | - |
| Incident scoliosis, n | 50 | 156 | 345 | 351 | 169 | - | - |
| Hazard ratio for incident scoliosis (95% CI) | | |  |  |  |  |  |
| Crude model | Reference | 0.81(0.59,1.12) | 0.81(0.60,1.09) | 0.73(0.54,0.98) | 0.70(0.51,0.96) | 0.93(0.88,0.99) | 0.014 |
| Adjusted model 1 | Reference | 0.78(0.57,1.07) | 0.78(0.58,1.04) | 0.69(0.52,0.94) | 0.61(0.44,0.83) | 0.90(0.86,0.96) | <0.001 |
| Adjusted model 2 | Reference | 0.79(0.57,1.08) | 0.79(0.58,1.06) | 0.71(0.53,0.96) | 0.62(0.45,0.86) | 0.91(0.86,0.96) | 0.001 |

A Cox proportional hazard model was conducted. Adjusted model 1, age, sex, ethnicity, household income,educational level, and Townsend deprivation index were adjusted for in this model; adjusted model 2, smoking status, alcohol status, diabetes, hypertension, and MET time per week additionally were adjusted for in this model.

CI, confidence interval.

**Supplementary Table 12. Sensitivity analyses regarding risk of incident scoliosis according to healthy sleep score**

| **Healthy sleep score** | **Participants, n** | **scoliosis, n** | **Adjusted HR (95% CI)** | ***P* value** | ***P* for trend** |
| --- | --- | --- | --- | --- | --- |
| **Sensitivity analysis 1: excluding scoliosis participants diagnosed within 1 year after baseline (N= 408783)** | | | | | |
| Per 1-point increment | - | - | 0.91(0.88,0.94) | <0.001 | <0.001 |
| 0-1 | 9932 | 93 | Reference | - |  |
| 2 | 46158 | 386 | 0.89(0.71,1.11) | 0.305 |  |
| 3 | 115067 | 858 | 0.81(0.65,1.00) | 0.054 | <0.001 |
| 4 | 150269 | 994 | 0.73(0.59,0.91) | 0.005 |  |
| 5 | 87357 | 558 | 0.67(0.54,0.84) | <0.001 |  |
| **Sensitivity analysis 2: excluding scoliosis participants diagnosed within 2 years after baseline (N=408684)** | | | | | |
| Per 1-point increment | - | - | 0.91(0.88,0.94) | <0.001 | <0.001 |
| 0-1 | 9930 | 91 | Reference | - |  |
| 2 | 46138 | 366 | 0.86(0.68,1.08) | 0.201 |  |
| 3 | 115040 | 831 | 0.80(0.64,1.00) | 0.046 | <0.001 |
| 4 | 150239 | 964 | 0.73(0.59,0.90) | 0.004 |  |
| 5 | 87337 | 538 | 0.66(0.53,0.83) | <0.001 |  |
| **Sensitivity analysis 3: excluding scoliosis participants diagnosed within 5 years after baseline (N=408291)** | | | | | |
| Per 1-point increment | - | - | 0.90(0.87,0.94) | <0.001 | <0.001 |
| 0-1 | 9917 | 78 | Reference | - |  |
| 2 | 46083 | 311 | 0.85(0.66,1.09) | 0.194 |  |
| 3 | 114933 | 724 | 0.81(0.64,1.02) | 0.071 | <0.001 |
| 4 | 150104 | 829 | 0.72(0.57,0.91) | 0.006 |  |
| 5 | 87254 | 455 | 0.64(0.50,0.82) | <0.001 |  |
| **Sensitivity analysis 4: excluding scoliosis participants diagnosed within 10 years after baseline (N=407183)** | | | | | |
| Per 1-point increment | - | - | 0.91(0.87,0.96) | <0.001 | <0.001 |
| 0-1 | 9874 | 35 | Reference | - |  |
| 2 | 45935 | 163 | 0.85(0.62,1.17) | 0.316 |  |
| 3 | 114592 | 383 | 0.82(0.61,1.11) | 0.201 | <0.001 |
| 4 | 149728 | 453 | 0.74(0.55,1.00) | 0.047 |  |
| 5 | 87054 | 255 | 0.66(0.49,0.89) | 0.007 |  |
| **Sensitivity analysis 5: Retain only white people, exclude individuals of other races. (N=377669)** | | | | | |
| Per 1-point increment | - | - | 0.90(0.87,0.93) | <0.001 | <0.001 |
| 0-1 | 8952 | 97 | Reference | - |  |
| 2 | 42343 | 375 | 0.82(0.66,1.03) | 0.083 |  |
| 3 | 106136 | 825 | 0.74(0.60,0.91) | 0.005 | <0.001 |
| 4 | 139035 | 949 | 0.66(0.54,0.82) | <0.001 |  |
| 5 | 81203 | 545 | 0.62(0.50,0.77) | <0.001 |  |
| **Sensitivity analysis 6: before March 11, 2020 (the start date of the COVID-19 pandemic) (N= 386575)** | | | | | |
| Per 1-point increment | - | - | 0.89(0.85,0.93) | <0.001 | <0.001 |
| 0-1 | 9198 | 56 | Reference | - |  |
| 2 | 43284 | 253 | 0.97(0.72,1.29) | 0.822 |  |
| 3 | 108323 | 519 | 0.82(0.62,1.08) | 0.153 | <0.001 |
| 4 | 142477 | 603 | 0.74(0.56,0.98) | 0.033 |  |
| 5 | 83293 | 332 | 0.66(0.50,0.88) | 0.005 |  |

A Cox proportional hazard model was conducted. All hazard ratios were calculated by adjusting for the following covariates: age, sex, ethnicity, household income, educational level, Townsend deprivation index, body mass index, smoking status, alcohol status, diabetes, hypertension, and MET time per week.

HR, hazard ratio; CI, confidence interval.

**Supplementary Table 13. Risk of scoliosis associated with healthy sleep score(Multiple interpolation of missing sleep data)**

|  | **Healthy sleep score** | | | | | **Per1-point increment** | ***P* for trend** |
| --- | --- | --- | --- | --- | --- | --- | --- |
|  | **0-1** | **2** | **3** | **4** | **5** |  |  |
| Participants, n | 12809 | 58463 | 141950 | 182646 | 104405 | - | - |
| Scoliosis, n | 135 | 528 | 1109 | 1271 | 675 | - | - |
| Hazard ratio for incident Scoliosis (95% CI) | | | | | | | |
| Crude model | Reference | 0.85(0.70,1.02) | 0.72(0.61,0.87) | 0.64(0.54,0.77) | 0.59(0.49,0.71) | 0.88(0.86,0.91) | <0.001 |
| Adjusted model 1 | Reference | 0.85(0.70,1.02) | 0.75(0.62,0.89) | 0.68(0.57,0.81) | 0.60(0.50,0.72) | 0.89(0.86,0.92) | <0.001 |
| Adjusted model 2 | Reference | 0.87(0.72,1.05) | 0.78(0.65,0.94) | 0.73(0.61,0.87) | 0.65(0.54,0.79) | 0.91(0.88,0.94) | <0.001 |

A Cox proportional hazard model was conducted. Adjusted model 1, age, sex, ethnicity, household income,educational level, and Townsend deprivation index were adjusted for in this model; adjusted model 2, body mass index, smoking status, alcohol status, diabetes, hypertension, and Total weekly activity time additionally were adjusted for in this model.

CI, confidence interval

**Supplementary Table 14. Comparison of results between the Fine-Gray model and our primary Cox model**

|  | **Healthy sleep score** | | | | | **Per1-point increment** | ***P* for trend** |
| --- | --- | --- | --- | --- | --- | --- | --- |
|  | **0-1** | **2** | **3** | **4** | **5** |  |  |
| Participants, n | 9939 | 46175 | 115094 | 150287 | 87375 | - | - |
| Scoliosis, n | 100 | 403 | 885 | 1012 | 576 | - | - |
| Death, n | 1515 | 5950 | 13377 | 15346 | 7091 | - | - |
| Hazard ratio for incident Scoliosis (95% CI) | | | | | | | |
| The original Cox model | Reference | 0.86(0.69,1.08) | 0.78(0.63,0.96) | 0.70(0.57,0.86) | 0.64(0.52,0.80) | 0.90(0.87,0.93) | <0.001 |
| Fine-Gray competing risk model | Reference | 0.87(0.70,1.08) | 0.79(0.64,0.97) | 0.70(0.57,0.87) | 0.65(0.53,0.81) | 0.90 (0.87,0.94) | <0.001 |

A Cox proportional hazard model was conducted. Adjusted model , age,sex, ethnicity, educational level, Townsend deprivation index, body mass index, smoking status, alcohol status, diabetes, hypertension, and MET time per week were adjusted for in this model

CI, confidence interval
