# Supplementary figures and images for: The relationship between healthy sleep patterns and the risk of scoliosis: a large prospective cohort study
Source: Front Neurosci. 2026 Jun 17;20:1839503. doi: 10.3389/fnins.2026.1839503 (PMC13320351; doi:10.3389/fnins.2026.1839503)

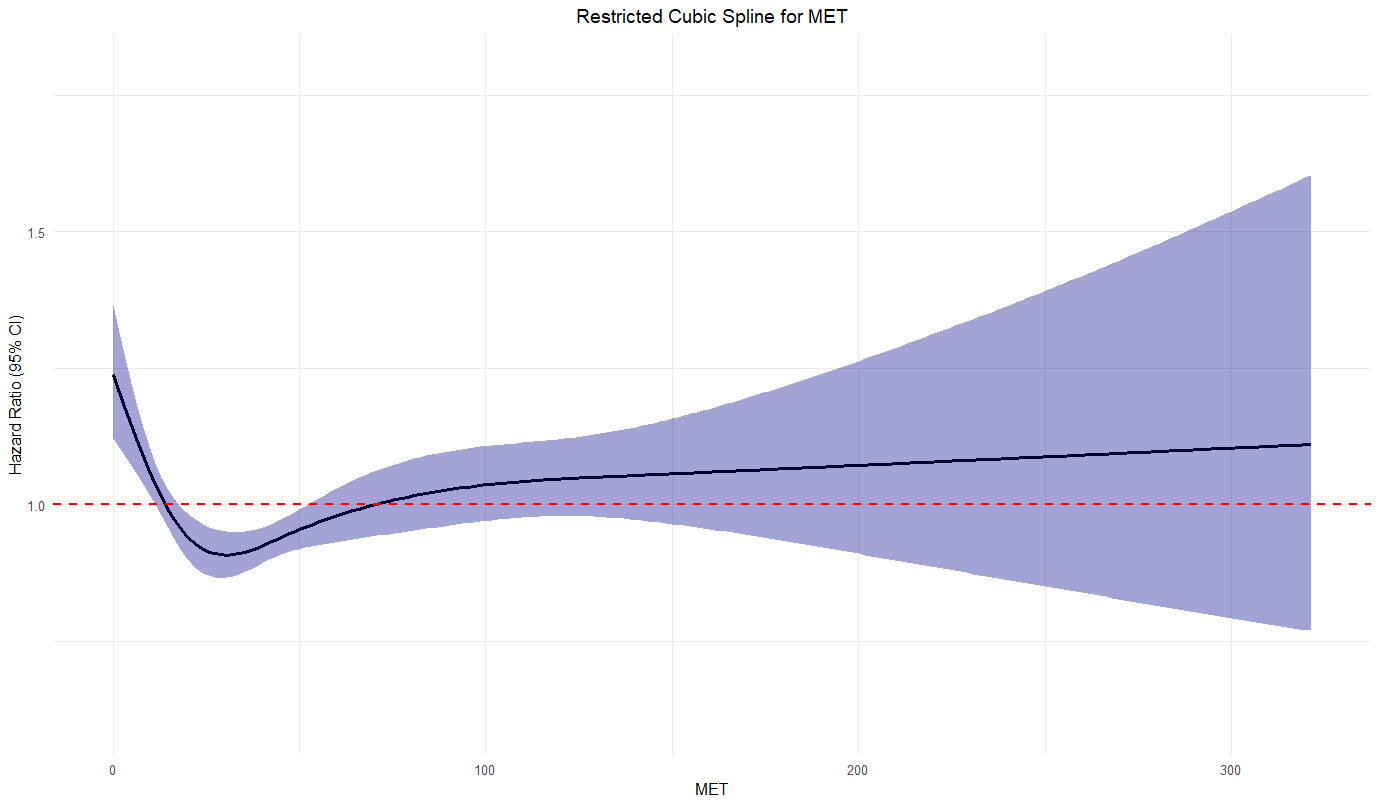

Supplement: Supplementary file 2 [file Image_1.tiff]
